# Effect of Angiotensin-Converting-Enzyme Inhibitor and Angiotensin II Receptor Antagonist Treatment on ACE2 Expression and SARS-CoV-2 Replication in Primary Airway Epithelial Cells

**DOI:** 10.3389/fphar.2021.765951

**Published:** 2021-11-19

**Authors:** Oghenemega Okoloko, Elizabeth R. Vanderwall, Lucille M. Rich, Maria P. White, Stephen R. Reeves, Whitney E. Harrington, Kaitlyn A. Barrow, Jason S. Debley

**Affiliations:** ^1^ Center for Immunity and Immunotherapies, Seattle Children’s Research Institute, Seattle, WA, United States; ^2^ Department of Pediatrics, Division of Pulmonary and Sleep Medicine, Seattle Children’s Hospital, University of Washington, Seattle, WA, United States; ^3^ Center for Global Infectious Disease Research, Seattle Children’s Research Institute, Seattle, WA, United States; ^4^ Department of Pediatrics, Division of Infectious Disease, Seattle Children’s Hospital, University of Washington, Seattle, WA, United States

**Keywords:** airway, epithelium, SARS—CoV—2, ACE2, angiotensin-converting eitzyme inhibitors, angiotensin II (A II) receptor antagonists, captopril, losartan

## Abstract

**Rationale:** SARS-CoV-2 gains entrance to airway epithelial cells (AECs) through binding of the viral spike protein to the angiotensin-converting enzyme 2 (ACE2) on the cell surface. However, ACE2 also converts angiotensin II into angiotensin-(1-7) and counterbalances the renin-angiotensin-aldosterone system, with resultant protective effects in the cardiovascular system. Some data suggest that two common antihypertension medications (angiotensin II receptor antagonists, ARBs; and angiotensin-converting-enzyme inhibitors, ACEIs) may increase ACE2 expression in heart and kidney cells, fueling debate about how these widely used medications may modulate SARS-CoV-2 infectivity and risk of COVID-19.

**Aim:** Determine whether exposure of bronchial AECs to the ARB losartan or the ACEI captopril modulate expression of ACE2 by AECs, SARS CoV2 replication, or expression of proinflammatory cytokines and type I and III interferon (IFN) responses.

**Methods:** Primary bronchial AECs from children and adults (*n* = 19; Ages 8–75 yrs) were differentiated *ex vivo* at an air-liquid interface to generate organotypic cultures. Cultures were treated with captopril (1 μM) or losartan (2 μM) with culture media changes starting 72 h before infection with SARS-CoV-2. In a biosafety level 3 (BSL-3) facility, cultures were infected with SARS-CoV-2 isolate USA-WA1/2020 at a multiplicity of infection (MOI) of 0.5. At 96 h following infection, RNA and protein were isolated. SARS-CoV-2 replication in cultures was assessed with quantitative PCR (qPCR). *ACE2, IL-6, IL-1B, IFNB1*, and *IFNL2* expression were assessed by qPCR.

**Results:** Neither captopril nor losartan treatment significantly changed *ACE2, IL-6, IL-1B, IFNB1*, or *IFNL2* expression by AECs as compared to SARS-CoV-2 infected AEC cultures without captopril or losartan treatment. At 96 h following infection, SARS-CoV-2 copy number/ng RNA was not significantly different between untreated AEC cultures, cultures treated with captopril, or cultures treated with losartan.

**Conclusion:** These findings suggest that at the level of the airway epithelium neither the ACEI captopril or ARB losartan significantly modify expression of the SARS-CoV-2 entry factor ACE2, nor does either medication increase replication SARS-CoV-2 replication. This *ex vivo* data is reassuring and is consistent with evolving clinical data suggesting ACEIs and ARBs do not increase the risk for poor prognosis with COVID-19 and may actually reduce the risk of COVID-19 disease.

## Introduction

SARS-CoV-2 has rapidly infected the human population, with over 245 million confirmed cases and over five million deaths worldwide by November 2021 (Medicine JHUa, 2021). SARS-CoV-2 gains entrance to airway epithelial cells (AECs) through binding of the viral spike protein to the angiotensin-converting enzyme 2 (ACE2) on the cell surface (Hoffmann et al., 2020). However, ACE2 also converts angiotensin II into angiotensin-(1-7) and counterbalances the renin-angiotensin-aldosterone system, with resultant protective effects in the cardiovascular system (Mascolo et al., 2021). Some data suggest that two common antihypertension medications (angiotensin II receptor antagonists, ARBs; and angiotensin-converting-enzyme inhibitors, ACEIs) may increase ACE2 expression in heart and kidney cells (Tikellis et al., 2003; Ferrario et al., 2005a; Ferrario et al., 2005b; Ocaranza et al., 2006; Yang et al., 2013; Zhang et al., 2014), fueling debate early in the COVID-19 pandemic about how these widely used medications might modulate SARS-CoV-2 infectivity and the risk of COVID-19.

In some animal studies ARBs and ACEIs were observed to increase ACE2 expression in some tissues (Tikellis et al., 2003; Ferrario et al., 2005a; Ferrario et al., 2005b; Ocaranza et al., 2006; Yang et al., 2013; Zhang et al., 2014), but the impact of these drugs on ACE2 expression in humans is mixed (Sriram and Insel, 2020), and ACE2 was found to be protective against pulmonary disease from SARS-CoV in mice (Altman et al., 2017). Questions about the effects of these medications on the infectivity and replication of SARS-CoV-2 and inflammatory response at the tissue/cell level, created widespread international controversy and concern early in the pandemic among patients and providers, with a paucity of mechanistic human data to inform decision making. In animal models, binding of the SARS-CoV spike protein to ACE2 leads to ACE2 downregulation (Altman et al., 2017), which in turn results in excessive production of angiotensin II by the related enzyme ACE. Decreased ACE2 may contribute to lung injury and respiratory failure, as ACE2 inhibits angiotensin II and prevents lung edema and inflammation (Imai et al., 2005; Kuba et al., 2005). Therefore, higher ACE2 expression from chronic treatment with ARBs or ACEIs, while seemingly counterintuitive given that ACE2 is the SARS-CoV-2 spike protein receptor, might actually protect patients against acute lung injury rather than putting them at higher risk of COVID-19 (Gurwitz, 2020). Furthermore, although ACEIs may increase ACE2 expression, they also decrease angiotensin-II (Düsing, 2016), and a low angiotensin-II state promotes formation of a complex between angiotensin II receptor I (ATR1) and the catalytic site of ACE2 (Deshotels et al., 2014), which could prevent binding of the SARS-Co-V2 spike protein to ACE2 and decrease infectivity. In contrast, although ARBs may increase ACE2 and angiotensin-II, effects that may facilitate SARS-CoV-2 infection and worsen lung injury, it has also been hypothesized that ARBs stabilize the ATR1-ACE2 complex (Deshotels et al., 2014) preventing spike protein binding to ACE2 and inhibiting SARS-CoV-2 infection and replication.

The aims of this study were to determine whether treatment of differentiated human bronchial AECs with the ARB losartan or the ACEI captopril modulate expression of ACE2 by AECs, SARS-CoV-2 replication, or the expression of inflammatory cytokines in the context of SARS-CoV-2 infection. We hypothesized that treatment of AEC cultures with losartan and/or captopril would increase expression of ACE2 and replication of SARS-CoV-2.

## Materials and Methods

### Differentiation of Primary Airway Epithelial Cells and Infection With SARS-CoV-2

Primary bronchial AECs from children ages 6–18 yrs (*n* = 12) and adults ages 60–75 yrs (*n* = 7) were differentiated *ex vivo* for 21 days at an air-liquid interface (ALI) on 12-well collagen-coated Corning® plates with permeable transwells in PneumaCult™ ALI media (Stemcell™) at 37°C in an atmosphere of 5% CO2 as we have previously described, producing an organotypic differentiated epithelial culture with mucociliary morphology (Lee et al., 2012; Lopez-Guisa et al., 2012; Iwanaga et al., 2013; Reeves et al., 2014; Reeves et al., 2015; Altman et al., 2017). All AECs from children were obtained under study #12490 approved by the Seattle Children’s Institutional Review Board, with investigations carried out following the rules of the Declaration of Helsinki of 1975. All adult AECs were purchased from Lonza®. In a Biosafety Level 3 (BSL-3) facility, organotypic AEC cultures were infected with SARS-CoV-2 isolate USA-WA1/2020 at a multiplicity of infection (MOI) of 0.5. Starting 72 h prior to SARS-CoV-2 infection, and with each media change for the duration of experiments, the ARB losartan (2 μM; Selleck Chem®) or ACEI captopril (1 μM; Selleck Chem®) were added to cell culture media. The concentrations of losartan (2 µM) and captopril (1 µM) were chosen after preliminary experiments in three primary AEC lines from children, using a range of concentrations for losartan at or greater than the concentration that inhibits 50% of the binding of angiotensin II for losartan (IC50 losartan; 20 nM), and range of concentrations for captopril near or greater than the concentration that inhibits 50% of ACE (IC50 captopril; 6 nM), using IC50 concentrations for each drug as reported by Selleck Chem®. Cell lysate and RNA were harvested, and AECs were prepped for immunohistochemistry (IHC), 96 h following infection with SARS-CoV-2.

To extract protein from the cell layer of SARS-CoV-2-infected AEC cultures, media was first removed from the basolateral chamber of transwells. Next, 100 µL of cold PBS was added to the apical surface of cultures and 1 ml was added to the basolateral chamber of cultures as a wash step. Next, 50 μL of RIPA buffer for protein extraction ready-to-use-solution (Sigma-Aldrich®, Product No. R0278) containing Triton X100 1% and SDS 0.1% was added to the apical surface of AECs and incubated for 15 min on ice. A pipet tip was then used to gently scratch each apical well in a crosshatch pattern to loosen AECs from the transwell membrane. Material was collected, centrifuged at 10,000 rpm a 4°C for 10 min, then supernatant containing isolated protein was collected.

RNA was isolated from SARS-CoV-2 infected AECs using a TRIzol™ Plus RNA Purification Kit (ThermoFisher). 500 uL of TRIzol™ was added to the apical surface of the transwell inserts, transwells were scraped in a crosshatch pattern to loosen the cells, then TRIzol™ was allowed to incubate for 5 min. Material was then added to 100 uL of chloroform (0.2 ml chloroform per 1 ml Trizol™) and incubated for 2–3 min, then centrifuged for 15 min at 12,000 × g at 4°C. 600 µL of the upper aqueous phase containing the RNA was transferred to a new tube to which an equal volume of 70% ethanol was added and the material vortexed. Thereafter, RNA was isolated following manufacturer protocols using appropriate TRIzol™ wash buffers and spin cartridges with final sample elution with RNase-free water (TRIzol™ Plus RNA Purification Kit, ThermoFisher®).

To measure viral replication in AEC cultures we performed quantitative polymerase chain reaction (qPCR) using the Genesig® Coronavirus Strain 2019-nCoV Advanced PCR Kit (Primerdesign®), with duplicate assays of harvested RNA from each SARS-CoV-2-infected AEC donor cell line, and quantification of viral replication as viral copy number/ng RNA. Concentration of RNA harvested from AECs was determined by Nanodrop®.

Expression of *ACE2, IL-1B, IL-6, IFNB1*, and *IFNL2* were measured by qPCR using Taqman® probes. ACE2 protein was measured in cell lysates using ELISA (R&D Systems®), with concentrations normalized to total protein levels in lysate (Pierce BCA Protein Assay Kit, ThermoFisher®).

### Immunofluorescence Confocal Microscopy of AECs

SARS-CoV-2 infected AEC transwell inserts were fixed at room temperature for 30 min by adding 600 µL of 10% formalin to the basolateral chamber and 200 µL of 10% formalin to the apical surface of AEC transwells. Both apical and basolateral surfaces of transwell inserts were washed 3 times with PBS. The tissue was treated for 30 min in blocking solution (5% FBS/0.05% Saponin in PBS) in the apical chamber. AECs were then incubated with primary antibodies for ACE2 (1:100; AF933, R&D Systems) and ATR1 (1:500; PA5-20812, Thermofisher) in blocking solution for 90 min at room temperature. Tissues were washed three times in PBS and then incubated with secondary antibodies (1:500; Donkey anti-Goat Alexafluor 488 and Donkey anti-Rabbit Alexafluor 568, Thermofisher) in blocking solution for 60 min at room temperature. Tissues were again washed 3 times in PBS. Transwell membranes were then carefully cut using an 8 mm biopsy punch and mounted to glass slides with mounting media (Vectashield). Images were acquired using confocal microscopy (TCS SP5, Leica).

### Statistical Analysis

Gene expression data and protein levels are presented as means +/− standard deviation (SD) when data were normally distributed, and as medians with interquartile range if one or more groups were not normally distributed. To determine if data was normally distributed the Kolmogorov-Smirnov test was used. To compare distributions across treatment conditions, one-way ANOVA was used if data was normally distributed, and the Friedman ANOVA test was used if data was non-normally distributed in one or more conditions. Post hoc comparisons between pairs of experimental conditions were made using Dunn’s multiple comparisons test (significance level set at *p* <0.05). Prism® 9.0 software (GraphPad Software Inc., San Diego, CA.) was used to analyze data. Relative gene expression was standardized using *GAPDH* as a non-regulated housekeeping gene. GenEx version 6 was used to analyze real-time qPCR results (MultiD Analyses AB, Göteborg, Sweden) based on methods described by Pfaffl (Pfaffl, 2001). Statistical significance was set at *p* <0.05.

## Results

Demographic and clinical characteristics of AEC donors are summarized in Table 1. Pediatric AEC donors (*n* = 12) had a mean age of 10.5 years and adult AEC donors (*n* = 7) had a mean age of 67 years. Nine of the pediatric donors were female and three of the adult donors were female. Two of the pediatric donors had a history of asthma. Of the adult donors, three were active smokers, four had been diagnosed with obesity (body mass index >30), four had been diagnosed with type 2 diabetes, and two had been diagnosed with hypertension.

**TABLE 1:** Demographics and clinical characteristics of AEC donors.

	**Pediatric AEC donors (** ***n*** ** = 12)**	**Adult AEC donors (** ***n*** ** = 7)**
Age (yrs., mean +/−SD)	10.5 +/− 2.0	67 +/− 4.9
Gender (female)	9 (75%)	3 (43%)
Active smoker	0 (0%)	3 (43%)
History of asthma	2 (17%)	0 (0%)
Obesity	0 (0%)	4 (57%)
Hypertension	0 (0%)	2 (29%)
Type 2 diabetes	0 (0%)	4 (57%)
Kidney disease	0 (0%)	unknown

**FIGURE 1:**
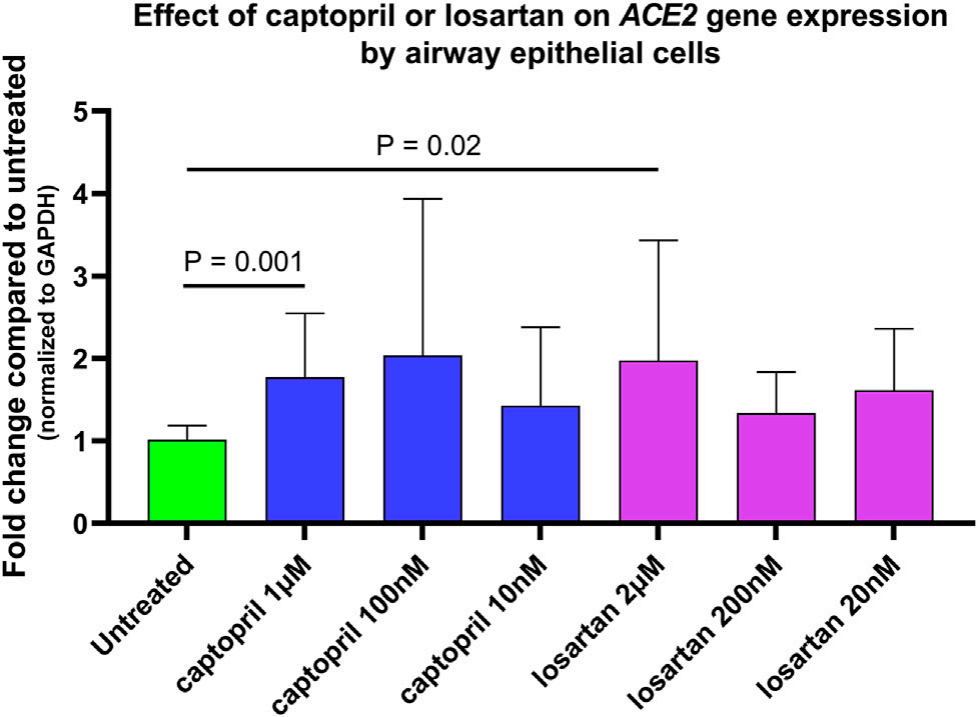
Relative *ACE2* gene expression (normalized to GAPDH expression) by untreated and captopril or losartan treated organotypic airway epithelial cell cultures. Cultures were treated with 1 µM, 100 nM, or 10 nM of captopril, and 2 µM, 200 nM, or 20 nM of losartan added to basolateral culture medium with each medium change for 7 days prior to RNA isolation. Each experimental condition was tested in triplicate wells of primary airway epithelial lines from three different children. Treatment of cultures with captopril or losartan led to significant increases in *ACE2* expression (Friedman ANOVA *p* = 0.0002) at concentrations of 1 µM of captopril (Dunn’s multiple comparison test for untreated vs. captopril treated cultures *p* = 0.001) and 2 µM of losartan (Dunn’s multiple comparison test for untreated vs. losartan treated cultures *p* = 0.02), however, there were not statistically significant differences in *ACE2* expression between the different concentrations of captopril or different concentrations of losartan tested.

In preliminary experiments, *ACE2* gene expression by untreated and captopril or losartan treated organotypic airway epithelial cell cultures was measured in three primary AEC lines from children. Cultures were treated with 1 µM, 100, or 10 nM of captopril, and 2 µM, 200, or 20 nM of losartan with each medium change for 7 days prior to RNA isolation. Treatment of cultures with captopril or losartan led to significant increases in *ACE2* expression (Figure 1; Friedman ANOVA *p* = 0.0002) at concentrations of 1 µM of captopril (Dunn’s multiple comparison test for untreated vs. captopril treated cultures *p* = 0.001) and 2 µM of losartan (Dunn’s multiple comparison test for untreated vs. losartan treated cultures *p* = 0.02), however, there were not statistically significant differences in *ACE2* expression between the different concentrations of captopril or different concentrations of losartan tested. In subsequent SARS-CoV-2 infection experiments concentrations of 1 µM of captopril and 2 µM of losartan were used.

**FIGURE 2:**
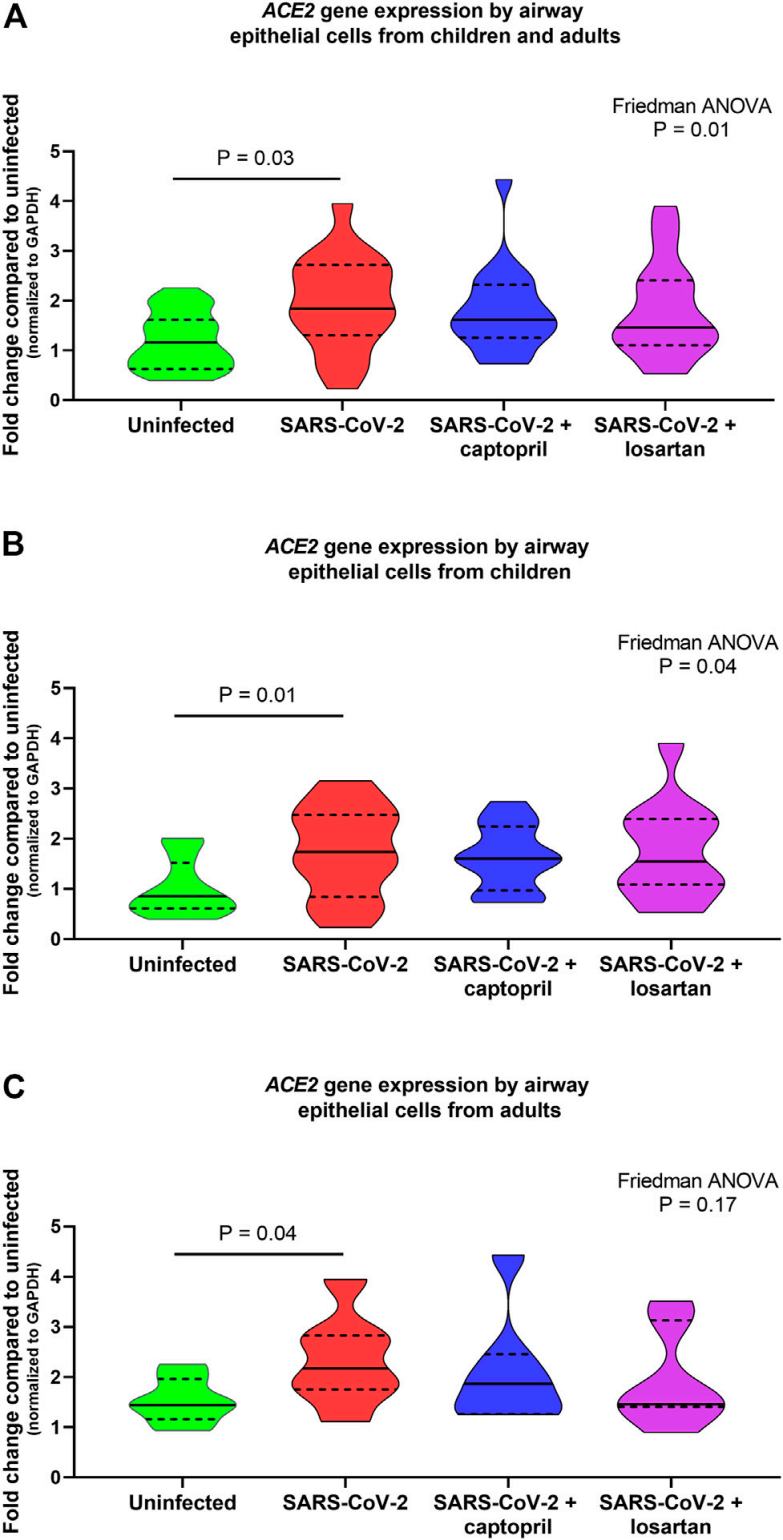
Relative gene expression of *ACE2* (normalized to GAPDH expression) by organotypic bronchial airway epithelial cell cultures from children (*n* = 12) and older adults (*n* = 7). *ACE2* expression was 1.6-fold greater in SARS-CoV-2 infected cultures as compared to uninfected cultures **(A)** children and adults combined, *n* = 19; Friedman ANOVA *p* = 0.01; Dunn’s multiple comparison test for uninfected vs. SARS-CoV-2 infected cultures *p* = 0.03), however, *ACE2* expression was not significantly different between SARS-CoV-2 infected cultures with or without treatment with captopril or losartan. **(B)** presents data from children separately (*n* = 12), wherein *ACE2* expression was 2-fold greater in SARS-CoV-2 infected cultures as compared to uninfected cultures (Friedman ANOVA *p* = 0.04; Dunn’s multiple comparison test for uninfected vs. SARS-CoV-2 infected cultures *p* = 0.01). *ACE2* expression was not significantly different between SARS-CoV-2 infected cultures with or without treatment with captopril or losartan. **(C)** presents data from adults separately (*n* = 7), wherein *ACE2* expression was 1.5-fold greater in SARS-CoV-2 infected cultures as compared to uninfected cultures (Friedman ANOVA *p* = 0.17; Dunn’s multiple comparison test for uninfected vs. SARS-CoV-2 infected cultures *p* = 0.03). *ACE2* expression was not significantly different between SARS-CoV-2 infected cultures with or without treatment with captopril or losartan. Median indicated by bold lines and quartiles indicated by dashed lines.

When data from cultures from children (*n* = 12) and older adults (*n* = 7) were analyzed together *ACE2* gene expression was 1.6-fold higher in SARS-CoV-2 infected AECs as compared to uninfected AECs (*n* = 19; Friedman ANOVA *p* = 0.01; Dunn’s multiple comparison test for uninfected vs. SARS-CoV-2 infected cultures *p* = 0.03; Figure 2A); however, *ACE2* expression was not significantly different between SARS-CoV-2 infected AEC cultures with or without treatment with captopril or losartan. In cultures from children alone (*n* = 12), *ACE2* expression was 2-fold greater in SARS-CoV-2 infected cultures as compared to uninfected cultures (Friedman ANOVA *p* = 0.04; Dunn’s multiple comparison test for uninfected vs. SARS-CoV-2 infected cultures *p* = 0.01; Figure 2B) and *ACE2* expression was not significantly different between SARS-CoV-2 infected cultures with or without treatment with captopril or losartan. In cultures from adults alone (*n* = 7), *ACE2* expression was 1.5-fold greater in SARS-CoV-2 infected cultures as compared to uninfected cultures (Friedman ANOVA *p* = 0.17; Dunn’s multiple comparison test for uninfected vs. SARS-CoV-2 infected cultures *p* = 0.03; Figure 2C) and *ACE2* expression was not significantly different between SARS-CoV-2 infected cultures with or without treatment with captopril or losartan.

**FIGURE 3:**
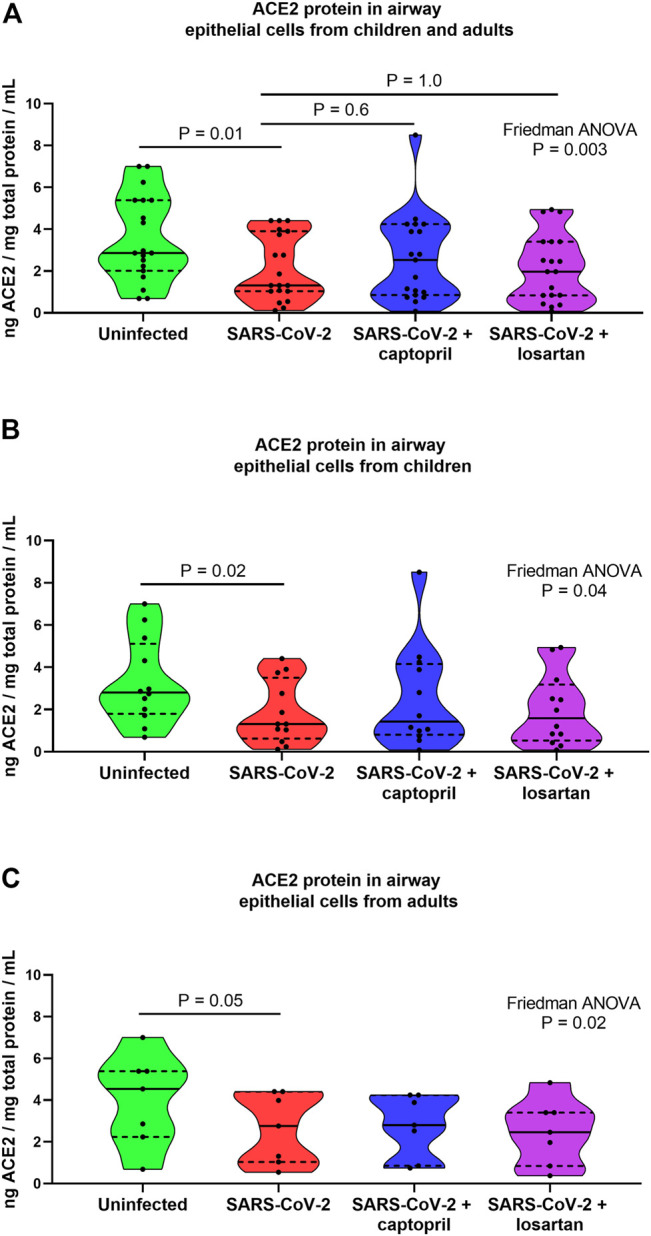
ACE2 protein concentrations in cell lysates from organotypic bronchial airway epithelial cell cultures from children (*n* = 12) and older adults (*n* = 7). ACE2 concentrations were normalized to total protein concentration in cell lysates. ACE2 protein concentrations were 2.2-fold lower in SARS-CoV-2 infected cultures as compared to uninfected cultures [**(A)** children and adults combined, *n* = 19; Friedman ANOVA *p* = 0.003; Dunn’s multiple comparison test for uninfected vs. SARS-CoV-2 infected cultures *p* = 0.01], however, ACE2 protein concentrations were not significantly different between SARS-CoV-2 infected cultures with or without treatment with captopril or losartan. **(B)** presents data from children separately (*n* = 12), wherein ACE2 concentrations were 2.2-fold lower in SARS-CoV-2 infected cultures as compared to uninfected cultures (Friedman ANOVA *p* = 0.04; Dunn’s multiple comparison test for uninfected vs. SARS-CoV-2 infected cultures *p* = 0.02). ACE2 concentrations were not significantly different between SARS-CoV-2 infected cultures with or without treatment with captopril or losartan. **(C)** presents data from adults separately (*n* = 7), wherein ACE2 concentrations were 1.6-fold lower in SARS-CoV-2 infected cultures as compared to uninfected cultures (Friedman ANOVA *p* = 0.02; Dunn’s multiple comparison test for uninfected vs. SARS-CoV-2 infected cultures *p* = 0.05). ACE2 concentrations were not significantly different between SARS-CoV-2 infected cultures with or without treatment with captopril or losartan. Median indicated by bold lines and quartiles indicated by dashed lines.

When data from cultures from children and older adults were analyzed together ACE2 protein concentrations were 2.2-fold lower in SARS-CoV-2 infected cultures as compared to uninfected AEC cultures (*n* = 19; Friedman ANOVA *p* = 0.003; Dunn’s multiple comparison test for uninfected vs. SARS-CoV-2 infected cultures *p* = 0.01; Figure 3A), however, there were not significant differences in ACE2 protein concentrations between SARS-CoV-2 infected AECs, SARS-CoV-2 infected AECs treated with captopril, or SARS-CoV-2 infected AECs treated with losartan (Figure 2). In cultures from children alone ACE2 concentrations were 2.2-fold lower in SARS-CoV-2 infected cultures as compared to uninfected cultures (Friedman ANOVA *p* = 0.04; Dunn’s multiple comparison test for uninfected vs. SARS-CoV-2 infected cultures *p* = 0.02; Figure 3B) and ACE2 concentrations were not significantly different between SARS-CoV-2 infected cultures with or without treatment with captopril or losartan. Similarly, in cultures from adults alone ACE2 concentrations were 1.6-fold lower in SARS-CoV-2 infected cultures as compared to uninfected cultures (Friedman ANOVA *p* = 0.02; Dunn’s multiple comparison test for uninfected vs. SARS-CoV-2 infected cultures *p* = 0.05; Figure 3C) and ACE2 concentrations were not significantly different between SARS-CoV-2 infected cultures with or without treatment with captopril or losartan.

**FIGURE 4:**
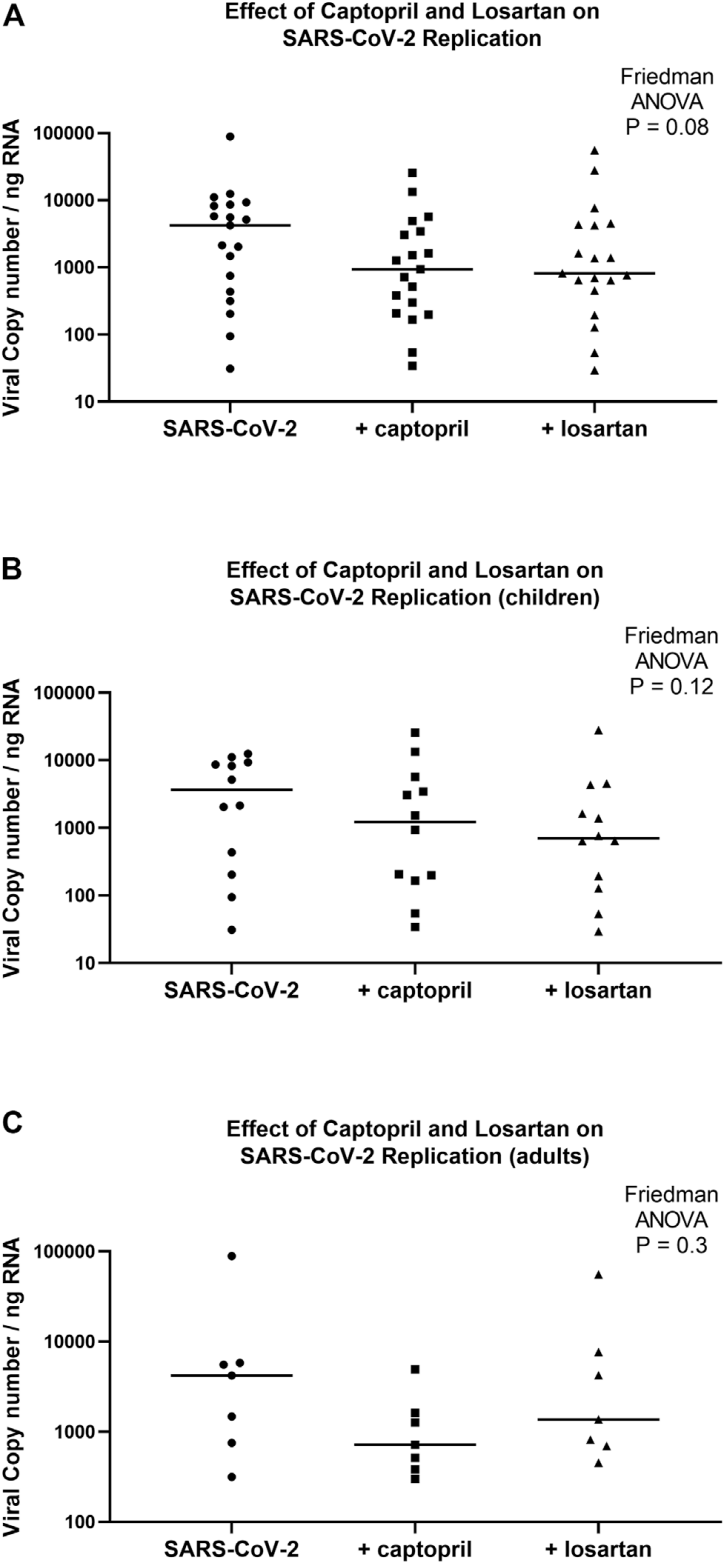
SARS-CoV-2 replication in organotypic bronchial airway epithelial cell cultures from children (*n* = 12) and older adults (*n* = 7) 96 h following infection of cultures with a multiplicity of infection (MOI) of 0.5. SARS-CoV-2 copy number by quantitative PCR normalized to total RNA concentration. SARS-CoV-2 copy number was not significantly different between captopril treated, losartan treated, and untreated cultures **(A)** (children and adults combined, *n* = 19; Friedman ANOVA *p* = 0.08). **(B)** presents data from children separately (*n* = 12), wherein SARS-CoV-2 replication was not significantly different between captopril treated, losartan treated, and untreated cultures (Friedman ANOVA *p* = 0.12). **(C)** presents data from adults separately (*n* = 7), wherein SARS-CoV-2 replication was not significantly different between captopril treated, losartan treated, and untreated cultures (Friedman ANOVA *p* = 0.3). Median indicated by bold lines.

Although there was a trend toward lower viral replication in captopril and losartan treated AEC cultures 96 h following infection (Friedman ANOVA *p* = 0.08), the median SARS-CoV-2 copy number/ng RNA was not significantly different between untreated AEC cultures (cultures from children and adults combined, *n* = 19; median 4,206, 95% CI 436–8,599), cultures treated with captopril (median 934, 95% CI 207–3,420; *p* = 0.15 for comparison to untreated), or cultures treated with losartan (median 813, 95% CI 452–4,309; *p* = 0.07 for comparison to untreated; Figure 4A). When pediatric and adult AEC cultures were analyzed separately, among pediatric AEC cultures (*n* = 12), the median SARS-CoV-2 copy number was not significantly different between untreated AEC cultures (median 3,633, 95% CI 204–9,281), cultures treated with captopril (median 1,227, 95% CI 167–5,700, *p* = 0.6 for comparison to untreated), or cultures treated with losartan (median 702, 95% CI 127–4,309; *p* = 0.08 for comparison to untreated; Friedman ANOVA *p* = 0.12; Figure 4B). Among adult AEC cultures (*n* = 7), the median SARS-CoV-2 copy number was also not significantly different between untreated AEC cultures (median 4,206, 95% CI 316–89,040), cultures treated with captopril (median 718, 95% CI 299–4,934, *p* = 0.2 for comparison to untreated), or cultures treated with losartan (median 1,365, 95% CI 452–55,580; *p* = 0.8 for comparison to untreated; Friedman ANOVA *p* = 0.3; Figure 4C).

**FIGURE 5:**
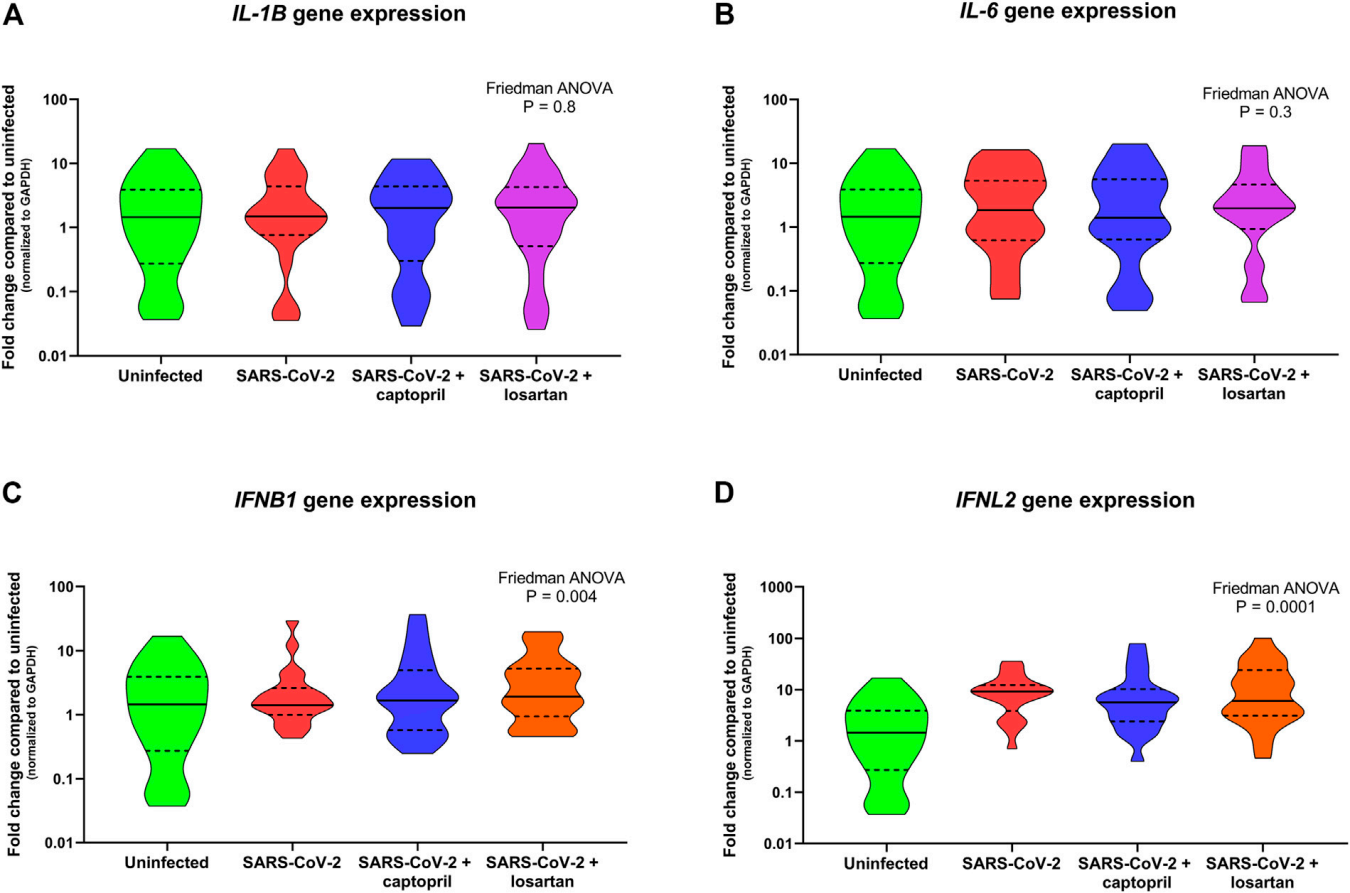
Relative gene expression of *IL-1B, IL-6, IFNB1*, and *IFNL2* (normalized to *GAPDH* expression) by organotypic bronchial airway epithelial cell cultures from children (*n* = 12) and older adults (*n* = 7). Expression of *IL-1B* was not significantly different between uninfected, SARS-CoV-2 infected, or SARS-CoV-2 infected cultures with or without treatment with captopril or losartan [**(A)** Friedman ANOVA *p* = 0.8]. Expression of *IL-6* was also not significantly different between uninfected, SARS-CoV-2 infected, or SARS-CoV-2 infected cultures with or without treatment with captopril or losartan [**(B)** Friedman ANOVA *p* = 0.3]. Expression of *IFNB1* and *IFNL2* were significantly greater by SARS-CoV-2 infected cultures compared to uninfected cultures [**(C)** Friedman ANOVA *p* = 0.004; **(D)** Friedman ANOVA *p* = 0.001]; however, there was no difference in *IFNB1* or *IFNL2* expression between SARS-CoV-2 infected cultures with or without treatment with captopril or losartan. Median indicated by bold lines and quartiles indicated by dashed lines.

**FIGURE 6:**
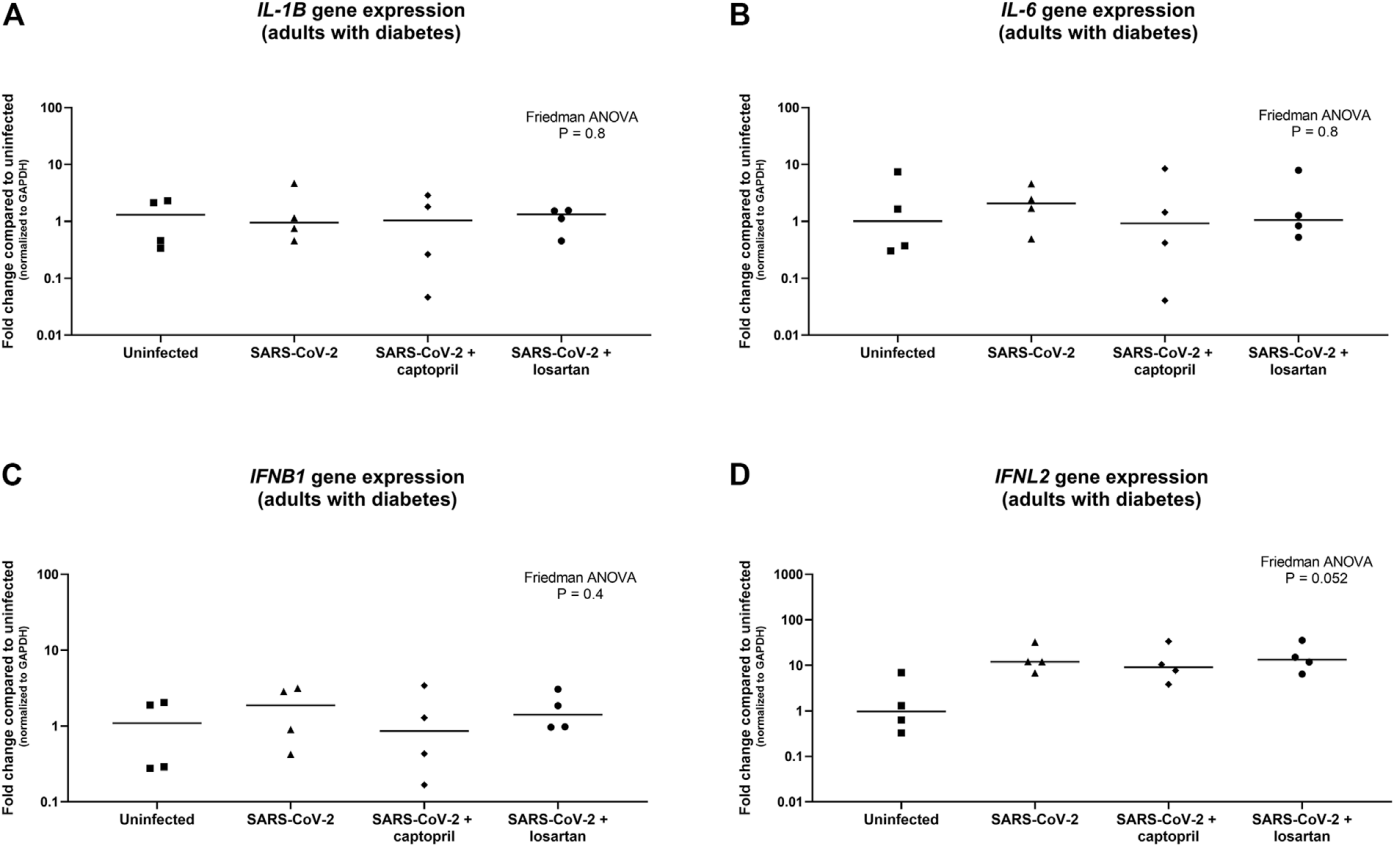
Relative gene expression of *IL-1B, IL-6, IFNB1*, and *IFNL2* (normalized to *GAPDH* expression) by organotypic bronchial airway epithelial cell cultures from adults with type 2 diabetes (*n* = 4). Expression of *IL-1B* was not significantly different between uninfected, SARS-CoV-2 infected, or SARS-CoV-2 infected cultures with or without treatment with captopril or losartan [**(A)** Friedman ANOVA *p* = 0.8]. Expression of *IL-6* was also not significantly different between uninfected, SARS-CoV-2 infected, or SARS-CoV-2 infected cultures with or without treatment with captopril or losartan [**(B)** Friedman ANOVA *p* = 0.8]. Expression of *IFNB1* was not significantly different between uninfected, SARS-CoV-2 infected, or SARS-CoV-2 infected cultures with or without treatment with captopril or losartan [**(C)** Friedman ANOVA *p* = 0.4], however, there was a trend toward greater expression of *IFNL2* by SARS-CoV-2 infected cultures compared to uninfected cultures [**(D)** Friedman ANOVA *p* = 0.052]. There was no difference in *IFNB1* or *IFNL2* expression between SARS-CoV-2 infected cultures with or without treatment with captopril or losartan. Median indicated by bold lines and quartiles indicated by dashed lines.

Expression of the pro-inflammatory cytokines *IL-1B* and *IL-6* were not significantly different between SARS-CoV-2 infected AECs, SARS-CoV-2 infected AECs treated with captopril, or SARS-CoV-2 infected AECs treated with losartan (Figures 5A,B). Similarly, expression of the type I and III interferons, *IFNB1* and *IFNL2*, were also not significantly different between SARS-CoV-2 infected AECs, SARS-CoV-2 infected AECs treated with captopril, or SARS-CoV-2 infected AECs treated with losartan (Figures 5C,D). We conducted a sub-analysis of data from the AEC cultures from the adults with type 2 diabetes (*n* = 4) and observed that expression of *IL-1B, IL-6, IFNB1, and IFNL2* were also not significantly different between SARS-CoV-2 infected AECs, SARS-CoV-2 infected AECs treated with captopril, or SARS-CoV-2 infected AECs treated with losartan (Figure 6 **)**.

**FIGURE 7:**
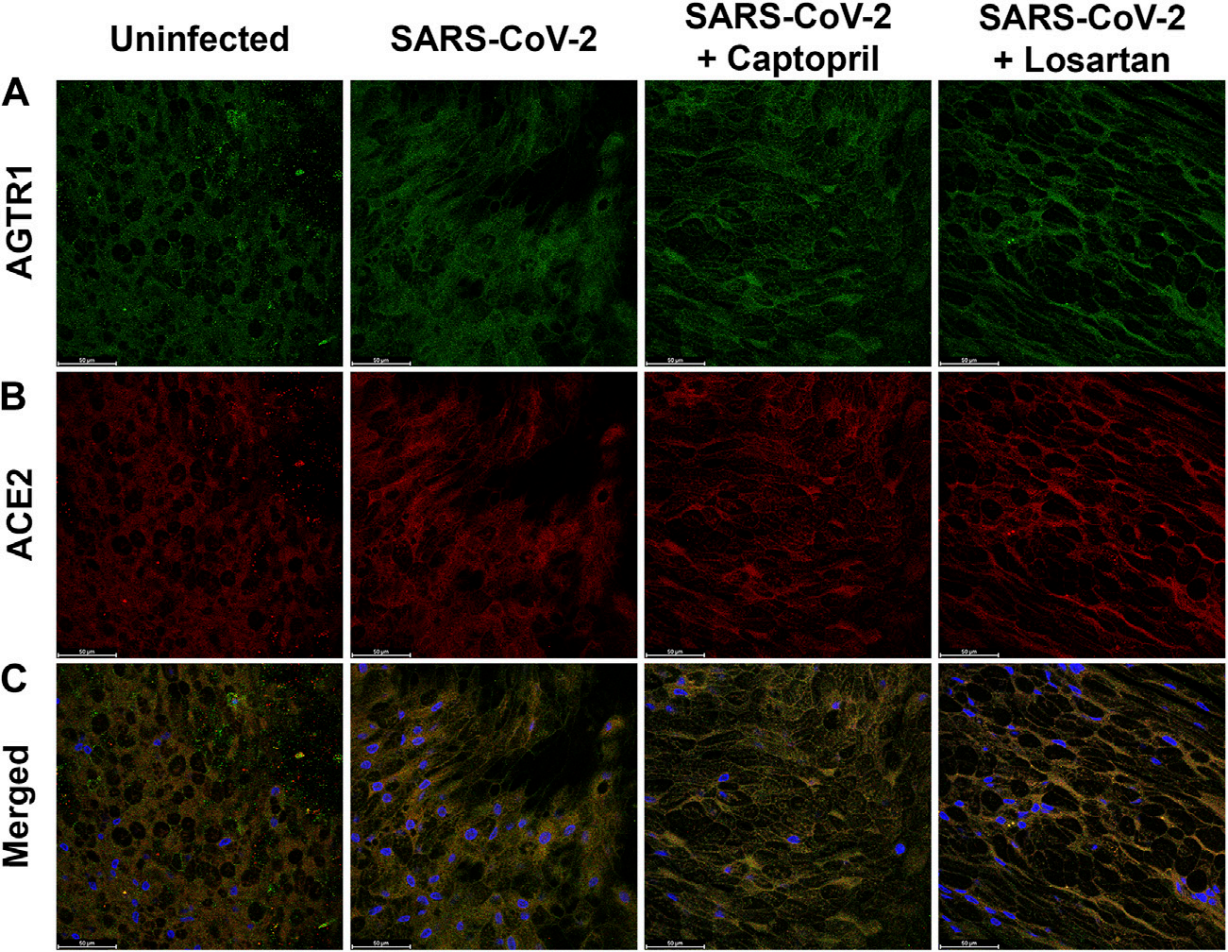
Immunofluorescence staining for ATR1 and ACE2 in primary differentiated airway epithelial cell cultures infected with SARS-CoV2 and treated with either captopril or losartan. Cultures were infected with SARS-CoV-2 for 96 h and a subset were treated with captopril (1 µM) or losartan (2 µM) throughout the 96-h infection. Depicted are representative confocal images obtained through the apical plane of differentiated airway epithelial cultures; ATR1 staining is shown in green **(A)**, ACE2 staining is shown in red **(B)**, and merged images including DAPI staining (blue) are shown in the **(C)**. No significant differences in staining pattern were observed between the groups (63x objective; Scale bars = 50 µm).

Given that ACEI treatment has been reported to promote formation of a complex between ATR1 and the catalytic site of ACE2, and that ARBs can stabilize the ATR1-ACE2 complex (Lee et al., 2012), we utilized IHC to visualize ACE2, ATR1, and co-localization of these proteins, in AEC cultures. We did not observe qualitative differences in ACE2 or ATR1 staining, or differences in the degree of co-localized staining of these proteins, between uninfected AECs, SARS-CoV-2 infected AECs, SARS-CoV-2 infected AECs treated with captopril, or SARS-CoV-2 infected AECs treated with losartan (representative images from *n* = 6 AEC lines; Figure 7).

## Discussion

We observed that SARS-CoV-2 infection of primary organotypic bronchial AEC cultures from children and adults led to modestly increased ACE2 gene expression yet modestly decreased ACE2 protein concentrations in AEC cultures. However, neither captopril nor losartan significantly altered ACE2 gene expression or protein concentrations in SARS-CoV-2 infected cultures. Furthermore, neither captopril or losartan significantly altered SARS-CoV-2 replication in primary bronchial AEC cultures, although we did observe a trend toward lower SARS-CoV-2 replication in captopril and losartan treated cultures. Finally, neither captopril nor losartan modified expression of the pro-inflammatory cytokines IL-6 or IL-1B, or changed expression of type I or III interferons, in SARS-CoV-2 infected AEC cultures. These findings suggest that at the level of the airway epithelium neither ACEIs or ARBs significantly modify expression of the SARS-CoV-2 entry factor ACE2, nor does either medication increase the replication of SARS-CoV-2 in differentiated airway epithelial cultures. These *ex vivo* data are reassuring and consistent with evolving clinical data suggesting ACEIs and ARBs do not increase the risk for poor prognosis with COVID-19.

Since the onset of the COVID-19 pandemic numerous studies (summarized in a recent meta-analysis (Grover and Oberoi, 2021)) have reported that co-existing conditions, including hypertension, cardiac diseases, cerebrovascular diseases, and diabetes, are more common among patients with COVID-19 who had severe illness, were admitted to the intensive care unit (ICU), received mechanical ventilation, or died, as compared to patients with mild illness. Early in the pandemic ACEI and ARB medications were hypothesized to increase the risk of severe COVID-19 (Diaz, 2020; Fang et al., 2020). This general hypothesis was informed by work published in the early 2000’s during and following the SARS-CoV and MERS-CoV epidemics, including studies wherein intravenous infusions of ACEI and ARB medications in experimental animals increased ACE2 receptor expression in the cardiopulmonary circulation (Ferrario et al., 2005a). Concern increased following publication of a descriptive analysis of 1,099 patients with laboratory-confirmed COVID-19 infections treated in China between December 2019 and January 2020 that reported that patients with COVID-19 infections and a history of hypertension or diabetes, conditions commonly treated with ACEIs or ARBs, suffered more severe disease outcomes (Guan et al., 2020). For much of the pandemic older patients suffered substantially greater morbidity and mortality from COVID-19 (Garg et al., 2021; Rapp et al., 2021). However, given that older adults, as compared to younger adults and children, are much more likely to have comorbidities including cardiovascular diseases, hypertension, diabetes, and chronic kidney disease for which they are treated with ACEIs or ARBs, associations between use of these medications and poor outcomes from COVID-19 may not be causative. Furthermore, Zhang et al. conducted a retrospective study of 1,128 adult patients with hypertension diagnosed with COVID-19, including 188 taking ACEIs or ARBs, and reported a lower risk of COVID-19 mortality in patients who received ACEI or ARB therapy compared to subjects who were not treated with these medications (Zhang et al., 2020). They also observed that compared to subjects taking other antihypertensive drugs, ACEI or ARB therapy was also associated with decreased mortality in patients with COVID-19 and coexisting hypertension.

Review of the literature regarding the effects of ACEIs or ARBs on *ACE2* expression in animal studies reveals a mixed picture. Ferrario et al. reported that treatment of mice with ACEIs or/and ARBs increased cardiac *ACE2* mRNA levels (Ferrario et al., 2005a). In contrast, Sriram et al. conducted a literature review of 12 different studies conducted using animal or human models and concluded that the data from these studies where inconsistent with the hypothesis of increased *ACE2* expression in response to AECI or ARB treatment (Sriram et al., 2020). Kriszta et al., published a comprehensive review of animal studies conducted since the early 2000’s and found that the majority of studies observed that ACEI/ARB treatment can cause ACE2 upregulation in tissues (Kriszta et al., 2021), however, they also reviewed evidence from several studies supporting the hypothesis that ACE2 activity inhibits acute lung injury (Imai et al., 2008; Gopallawa and Uhal, 2014) through formation of Angiotensin (1–7), which through its interaction with the Mas receptor blocks detrimental effects of Angiotensin II induced oxidative stress, inflammation, and tissue injury by Angiotensin II mediated by the AT1 receptor, suggesting that these drugs could be protective against severe morbidity or mortality from COVID-19. However, Kriszta et al. concluded that prospective well designed randomized trials are required to definitely answer whether ACEIs or ARBs increase or decrease the risk of morbidity and mortality in COVID-19 (Kriszta et al., 2021). The lack of consensus regarding the impact of ACEI or ARB treatment on ACE2 levels on the cell surface has led to prolonged concerns about the safety of continued administration of these medications to COVID-19 patients.

Systemic immune dysregulation with production of elevated levels of pro-inflammatory cytokines have been reported to be associated with severe COVID-19 (Conti et al., 2020; Herold et al., 2020; Della-Torre et al., 2021; Jones and Hunter, 2021; Saji et al., 2021; Utrero-Rico et al., 2021; Yang et al., 2021). Two sentinel cytokines associated with this “cytokine storm” in COVID-19, IL-6 and IL-1B, are expressed by not only immune cells but also by the airway epithelium itself (Hastie et al., 1996; Stríz et al., 1999; Nagarkar et al., 2012; Martinez-Nunez et al., 2014; Jevnikar et al., 2019). Given the hypothesis that ACE2 activity may inhibit inflammation and lung injury by increasing angiotensin (1–7) which through its interactions with the Mas signaling cascade may dampen detrimental effects of Ang II induced oxidative stress and inflammation (Imai et al., 2008; Gopallawa and Uhal, 2014), we assessed whether ACEIs or ARBs impacted expression of either of these cytokines in SARS-CoV-2 infected primary AEC cultures. Surprisingly we observed that at the level of the epithelium SARS-CoV-2 did not significantly impact expression of either *IL-6* or *IL-1B*. Furthermore, neither captopril or losartan changed expression of *IL-6* or *IL-1B* in AECs infected with SARS-CoV-2. We also conducted a sub-analysis of data from AEC cultures from the four adults with type 2 diabetes and observed that the overall impacts of captopril and losartan on IL-1B, IL-6, IFNB1, and IFNL2 expression by SARS-CoV-2 infected AECs from diabetic donors were similar to those observed in the analysis from the full dataset, however, this analysis was limited by a small sample size. However, together these data suggest that the contribution of IL-6 and IL-1B to cytokine storm in severe COVID-19 is the result of systemic immune dysregulation downstream of the epithelium. Furthermore, ACEI/ARB treatment of organotypic AEC cultures does not impact expression of these pro-inflammatory cytokines.

The type I and III interferons, IFNβ and IFBλ, are critical elements of the innate immune response of mucosal cells to viral infection that trigger expression of an array of interferon stimulated genes that work to both limit viral propagation locally and activate adaptive immune responses (Vareille et al., 2011; Altman et al., 2018). The role of interferon responses in COVID-19 appears complex. On the one hand, elevated systemic IFN responses appear to be associated with severe COVID-19 (Lee and Shin, 2020; Zhou et al., 2020; Zhu et al., 2020), while there is also evolving evidence that SARS-CoV-2 may evade type I and III IFN responses at the level of the airway epithelium (Totura and Baric, 2012; Park and Iwasaki, 2020; Sa Ribero et al., 2020) which may allow the virus to replicate more efficiently potentially setting the stage for a later dysregulated immune response systemically. Given the importance of IFN responses to SARS-CoV-2 we sought to determine if ACEI or ARB medications had any impact on IFN responses. We observed that SARS-CoV-2 infection of primary AEC cultures increased expression of *IFNB1* and *IFNL2*; however, neither captopril nor losartan had any effect on expression of these interferons by AECs. Of note, our observation of an increase in ACE2 gene expression with SARS-CoV-2 infection is consistent with ACE2 being an interferon stimulated gene (ISG) as we and other groups have reported (Murphy et al., 2020; Ziegler et al., 2020). However, we also observed a decrease is ACE2 protein concentration in SARS-CoV-2 infected cultures, which we postulate may be explained by shedding or internalization and degradation of cell surface ACE2 with SARS-CoV-2 infection.

We are not aware of other studies to date that have assessed the impact of ACEIs or ARBs on ACE2 expression and SARS-CoV-2 replication in organotypic cultures using primary human airway epithelial cells, the site of initial infection of the respiratory tract by SARS-CoV-2. However, there are several limitations of our primary airway epithelial model system. First, our *ex vivo* system lacks interaction with immune cells and the complex immune responses that occur *in vivo* in the context of COVID-19, and therefore we cannot assess how ACEIs or ARBs interact with the systemic immune response to SARS-CoV-2 beyond the level of the airway epithelium. Second, ACEI and ARB medications have significant impacts on cardiopulmonary physiology which also cannot be assessed in our model system. Acute lung injury has been shown to significantly downregulate expression of *ACE2* in the lung, and ACE2 is a sentinel enzyme that regulates the renin–angiotensin system (Imai et al., 2005). Prior *in vivo* murine studies demonstrated that although ACEIs or ARBs may increase *ACE2* expression (Tikellis et al., 2003; Ferrario et al., 2005a; Ferrario et al., 2005b; Ocaranza et al., 2006; Yang et al., 2013; Zhang et al., 2014), ACE2 was protective against acute respiratory distress and lung failure (Imai et al., 2005). Although we did not observe an impact of ACEI or ARB medications on ACE2 gene expression or protein concentration in SARS-CoV-2 infected AEC cultures our study cannot model the impact of these medications on alveolar or endothelial cells or the impact of these medications on *in vivo* cardiopulmonary physiology.

We are not aware of data in humans regarding bronchial airway epithelial concentrations of losartan or captopril achieved after typical oral dosing. Another limitation of our study design is that the concentrations of these drugs used in this study may be different than what is actually present in the bronchial epithelium in a patient taking either of these medications. However, we believe that 2 µM losartan and 1 µM of captopril are likely greater than tissue concentrations achieved with oral dosing and therefore would tend to bias our results toward observing an effect of these medications on ACE2 gene expression and protein concentrations as well as SARS-CoV-2 replication. Additional limitations include the small number adults with type 2 diabetes in this study and the lack of subjects with known kidney disease, two patient populations where ACEIs and ARBs are commonly prescribed. Finally, although our results refuted our *a priori* hypothesis that ACEIs or ARBs would increase viral replication, we observed a trend toward lower SARS-CoV-2 replication in epithelial cultures treated with captopril or losartan. It is possible that with a larger sample size this trend toward a reduction in viral replication might have reached statistical significance.

In conclusion, using organotypic bronchial AEC cultures from children and adults we found that neither the ACEI captopril nor the ARB losartan significantly altered *ACE2* gene expression or ACE2 protein concentrations in the context of SARS-CoV-2 infection, nor did they increase replication of SARS-CoV-2 in the epithelium. Furthermore, we did not observe an impact of these medication on the pro-inflammatory cytokines IL-6 or IL-1B, or on the innate type I or III interferon response to SARS-CoV-2 by the epithelium. These data from a highly unique primary airway epithelial *ex vivo* model system are reassuring in that they demonstrate that ACEIs and ARBs, which are used by millions of people worldwide, do not increase the risk of SARS-CoV-2 replication at the site of initial infection. These results are also consistent with growing observational clinical data suggesting that these medications do not increase the risk for poor prognosis with COVID-19.

## Data Availability

The original contributions presented in the study are included in the article. Datasets used and/or analyzed during the current study are available from the corresponding author upon reasonable request.
